# First report of interspecific transmission of sarcoptic mange from Iberian ibex to wild boar

**DOI:** 10.1186/s13071-021-04979-w

**Published:** 2021-09-19

**Authors:** Marta Valldeperes, Barbara Moroni, Luca Rossi, Jorge Ramón López-Olvera, Roser Velarde, Anna Rita Molinar Min, Gregorio Mentaberre, Emmanuel Serrano, Samer Angelone, Santiago Lavín, José Enrique Granados

**Affiliations:** 1 Wildlife Ecology & Health group (WE&H) and Servei d’Ecopatologia de Fauna Salvatge (SEFaS), Departament de Medicina i Cirurgia Animals, Universitat Autònoma de Barcelona (UAB), Bellaterra, Spain; 2 Dipartimento di Scienze Veterinarie, Universitá di Torino, Grugliasco, Turin, Italy; 3 Wildlife Ecology & Health group (WE&H) and Departament de Ciència Animal, Escola Tècnica Superior d’Enginyeria Agrària (ETSEA), Universitat de Lleida (UdL), Lleida, Spain; 4 Institute of Evolutionary Biology and Environmental Studies (IEU), University of Zürich, Zürich, Switzerland; 5 Espacio Natural de Sierra Nevada and Wildlife Ecology & Health Group (WE&H), Pinos Genil, Granada, Spain

**Keywords:** *Capra pyrenaica*, Cross-transmission, *Sarcoptes scabiei*, Spain, *Sus scrofa*

## Abstract

**Background:**

Sarcoptic mange is a globally distributed parasitic disease caused by the burrowing mite *Sarcoptes scabiei*. This mite has a certain degree of host specificity, although interspecific transmission can occur among phylogenetically related species or through prey–predator mediated exposure. In 2018, a wild boar (*Sus scrofa*) with lesions compatible with sarcoptic mange was hunted in Ports de Tortosa i Beseit Natural Park (PTB, north-eastern Spain), where an active epizootic outbreak of sarcoptic mange is affecting Iberian ibexes (*Capra pyrenaica*) since 2014.

**Methods:**

A complete necropsy, skin scrapings and skin digestions with hydroxide potassium were performed to confirm the diagnosis. Routine histopathological analysis, toluidine blue staining and immunohistochemistry were used to characterize the lesions and the inflammatory infiltrate. Finally, 10 specific *S. scabiei* microsatellites were molecularly genotyped through polymerase chain reactions in mites obtained from the affected wild boar. For phylogenetic comparison, mites obtained from sympatric Iberian ibexes and allopatric wild boars and Iberian ibexes from southern Spain were analysed.

**Results:**

*Sarcoptes scabiei* was visually and molecularly identified in the infested wild boar from PTB, causing skin lesions with dermal inflammatory infiltrate rich in T and B cells, which indicate an adaptive immune response. Three *S. scabiei* genetic clusters were identified: one included mites from southern Iberian ibexes, another included mites from southern wild boars, and a third one distinctively grouped the wild boar from PTB with the sympatric ibexes.

**Conclusions:**

To the authors’ knowledge, this is the first reported case of sarcoptic mange in wild boar in Spain and the first documented case of *S. scabiei* cross-transmission from a wild ruminant host to a wild boar. The wild boar presented an ordinary scabies type reaction, which is typical of the self-limiting infestations reported in other cases of interspecific transmission.

**Graphical abstract:**

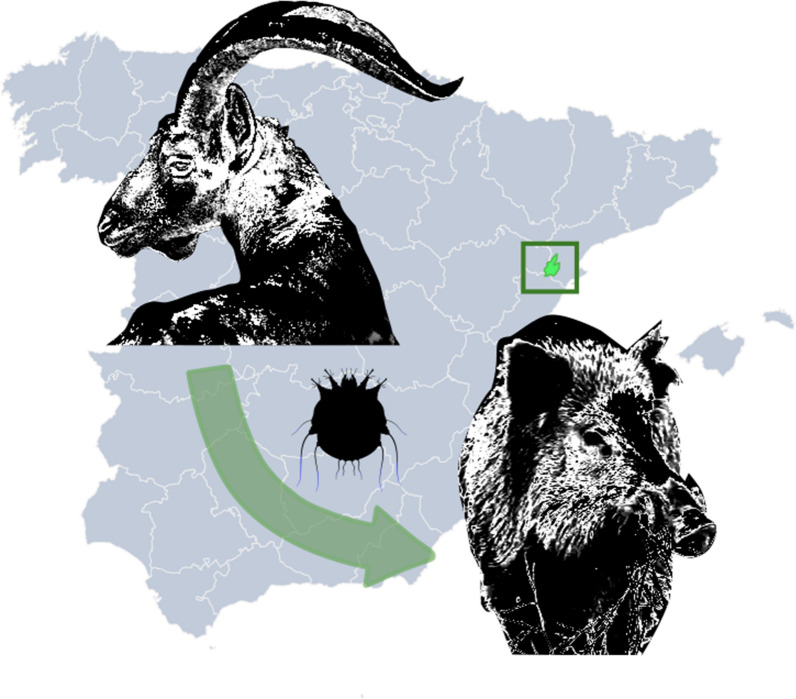

**Supplementary Information:**

The online version contains supplementary material available at 10.1186/s13071-021-04979-w.

## Background

Sarcoptic mange is an emerging and re-emerging parasitic disease of human and wildlife health concern caused by the burrowing mite *Sarcoptes scabiei* [1–3]. It has been described in more than 100 mammal species, causing wildlife population declines and livestock economic losses in a number of them due to difficulties in controlling the disease, specifically in wild free-ranging ungulate populations [3, 4]. *Sarcoptes scabiei* is considered a single species with a wide range of host-specific variants or lineages based on the host where they are collected, such as the carnivore or ungulate variants [5–7]. However, there is an ongoing debate about the host and geographic specificity and the potential of interspecific transmission of the mite [8]. Sarcoptic mange has been defined as an emerging panzootic in wildlife due to its ongoing global transmission and sustained spread among areas and wildlife species. Understanding the susceptible hosts and transmission pathways for the mite and each one of the variants should allow us to establish the potential host communities for *S. scabiei* and the consequent effects on wildlife conservation, livestock health and zoonotic risk [7]. Attempts at clustering *S. scabiei* by host species and geographical localisation have ended up with controversial results, at least partly due to the variability of molecular markers used [9–12]. However, microsatellites have become the most used and accepted molecular markers for the identification of *Sarcoptes* host-taxon clusters [10–14] and in forensic investigations of scabietic traded animals [15] to trace the origin of outbreaks and mite genetic population distances.

Even though sarcoptic mange transmission is mostly direct, indirect transmission among different host species is also feasible, as mites may survive off-host for up to 19 days (depending on temperature and relative humidity) and thus encounter other potential hosts [6, 16, 17]. Descriptive and experimental studies on cross-transmission have generated heterogeneous results, hence the host specificity of *S. scabiei* strains remains unclear [6, 7, 12, 16–20]. Spill-over events have been mostly reported in phylogenetically related species or prey–predator relationships, with other interspecific transmissions between phylogenetically distant species mostly resulting in self-limiting infections [7, 14, 21–25].

Sarcoptic mange, though deeply investigated in domestic pig due to its negative impact on productivity and as a model for human scabies [26–31], has been poorly studied in wild boar (*Sus scrofa*), where this skin disease is probably underreported [1, 10, 32]. Sarcoptic mange is present in swine production of Spain [29, 30], and wild boar populations are increasing in Europe, including urban, peri-urban and humanised areas [33–37]. However, in spite of external inspection of all the wild boars hunted and the existence of a national wildlife health surveillance programme [38], no macroscopic clinical cases of sarcoptic mange have been reported to date in wild boar in Spain. Nevertheless, the detection of a low seroprevalence against *S. scabiei* amongst wild boars in Spain (1.2%) [32] suggests that the mite has come in contact with or even circulated in local wild boar populations. Apparently, such contact has not led to widespread clinical disease or epidemic outbreaks, which probably would have been detected through the aforementioned wildlife health surveillance, although some subclinical or clinical cases may have occurred unnoticed. Conversely, sarcoptic mange outbreaks have been widely studied in the Iberian ibex (*Capra pyrenaica*) populations from the Iberian Peninsula since 1987 due to the impact on host demography and the related economy [39, 40]. The latest of these outbreaks is affecting the Iberian ibex population of Ports de Tortosa i Beseit Natural Park (PTB) in north-eastern Spain since December 2014 [41, 42]. Wild boars and Iberian ibexes coexist in natural scenarios of the Iberian Peninsula but have different habitat preferences, and pathogen cross-transmission between these two ungulate species is rare, even in areas of high pathogen prevalence [43]. These factors pose a challenge for sarcoptic mange transmission between both species, particularly considering the relatively short survival of *S. scabiei* off the host.

This study aims to describe the first clinical cases of sarcoptic mange in wild boars from the Iberian Peninsula and to identify the possible source of the infection by comparing mites from different wild boar and sympatric Iberian ibex populations in Spain using microsatellites as molecular markers.

## Methods

### Animals

**Fig. 1:**
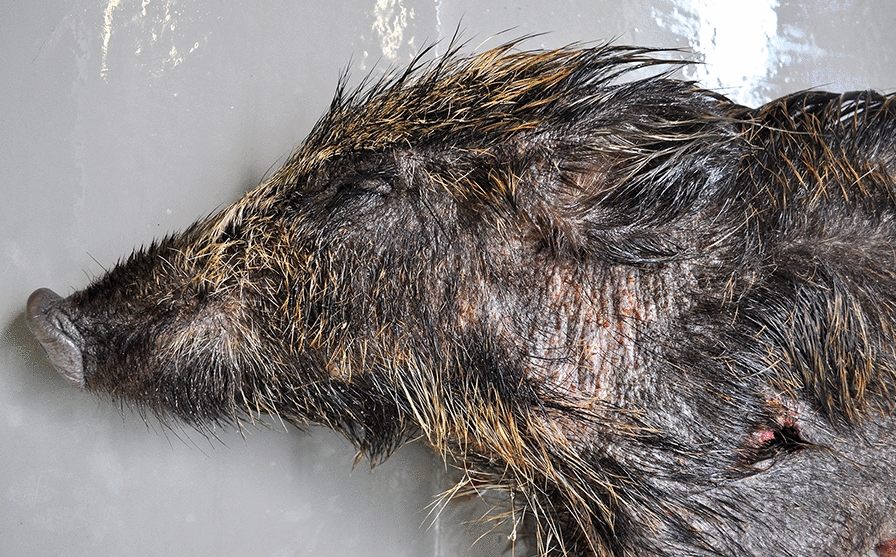
Macroscopic lesions of sarcoptic mange of the wild boar study case (wildb1), with alopecia on the head and neck, mild to moderate skin thickening, papules and crusting

**Fig. 2:**
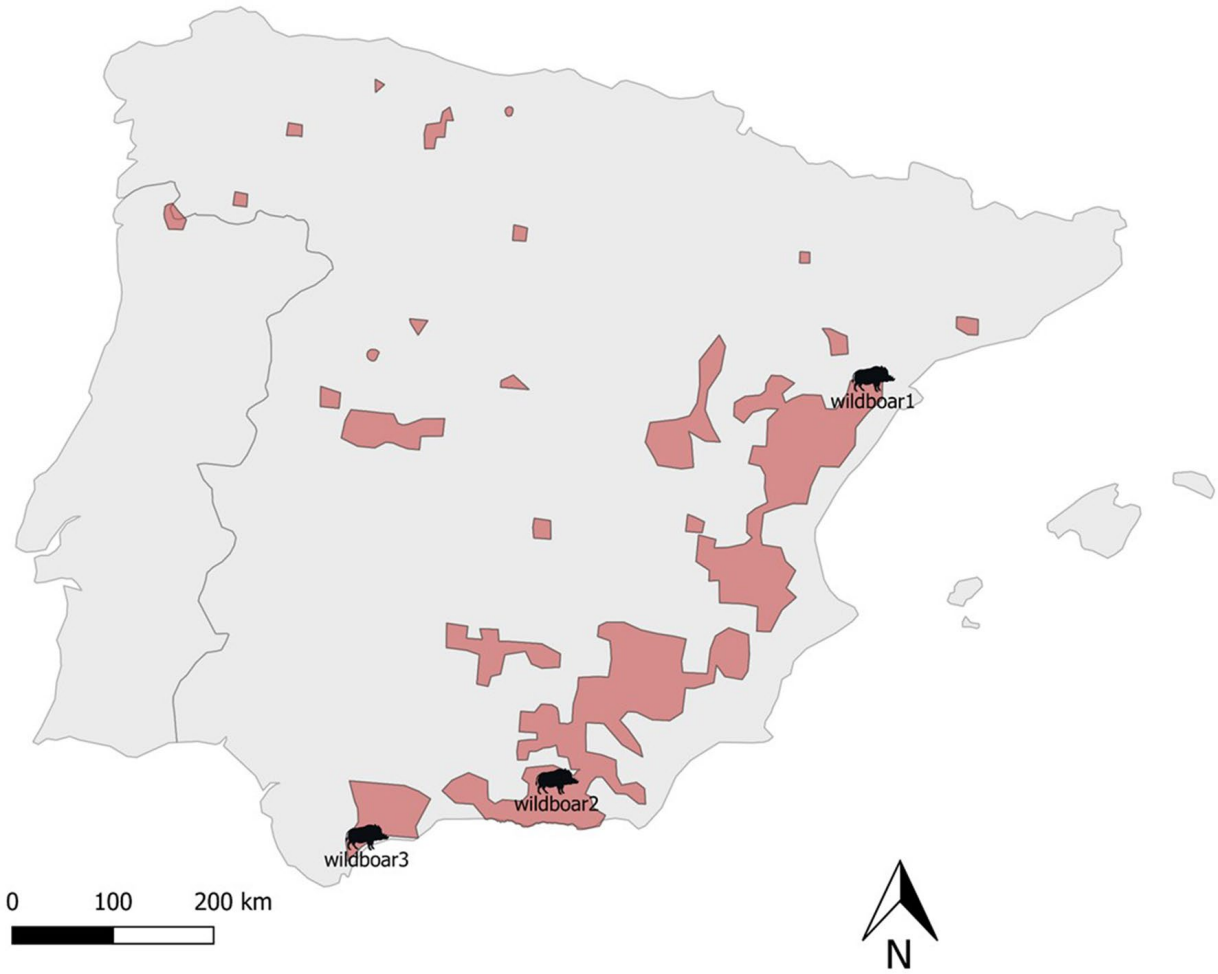
Map of the Iberian Peninsula showing the distribution of *Capra pyrenaica* (light red) and the origin of the three scabietic wild boars included in this study. The shapefiles of Iberian ibex distribution were obtained from the Red List of Threatened Species of the International Union for Conservation of Nature (https://www.iucnredlist.org), and the map was prepared using QGIS software 3.2.0 “Bonn” (http://qgis.osgeo.org)

On 29 October 2018, an 8-month-old female wild boar (wildb1) was hunted in a private hunting area (40°52′44.7″ N, 0°17′23.3″ E) in Arnes (Tarragona, north-eastern Spain), within the PTB. The wild boar had moderate skin lesions consistent with sarcoptic mange [44] (Fig. 1) and was submitted to the Servei d’Ecopatologia de la Fauna Salvatge (SEFaS) of the Universitat Autònoma de Barcelona (UAB) for post-mortem examination as well as histopathology, immunohistochemistry, inflammatory cell and molecular analyses. Two additional wild boars, also with moderate skin lesions compatible with sarcoptic mange from southern Spain, were hunted in 2014 in the Lanteira (Granada), within the regular management plan of the species in the National Park of Sierra Nevada (wildb2), and found alive as an orphan piglet in 2017 in Sierra Bermeja (Málaga, wildb3), respectively. The mites obtained from these two wild boars and from 23 sympatric Iberian ibexes were used for molecular analysis to establish phylogenetic relationships (Fig. 2). Shapefiles of Iberian ibex distribution obtained from the Red List of Threatened Species of the International Union for Conservation of Nature [45] were used to construct a map using QGIS software 3.2.0 “Bonn” [46]. Skin samples from an apparently healthy wild boar hunted within the regular management of the species were collected as control for *S. scabiei* identification and the histopathological and immunohistochemistry analyses.

### Post-mortem examination

A complete systematic necropsy following a standardized protocol was performed on wildb1, recording macroscopic lesions. Deep skin scrapings from the area of transition between healthy and affected skin were obtained using a sterile scalpel and evaluated under an optic stereomicroscope (Leica EZ4) at ×35. A blood sample was collected from the cavernous sinus [47]. Tonsil, submandibular and retropharyngeal lymph nodes, spleen, lung and skin were also collected in microtubes and kept at −20 °C. Lung tissue was submitted for routine microbiological culture. Additional tissue samples including skin, lung, cervical lymph nodes, liver, spleen, kidney, skeletal muscle, heart and brain were fixed in neutral-buffered 4.5% formalin for at least 24 h, processed and embedded in paraffin for routine histopathological microscopic examination. Three additional wildb1 skin samples of 2.5 × 2.5 cm were collected from the margins of the lesions containing healthy and damaged skin, and processed in a 5% potassium hydroxide (KOH) solution overnight at 43 °C. The digestion products of the three wild boars affected by sarcoptic mange (wildb1, wildb2 and wildb3) and the control wild boar were inspected using an optic stereomicroscope (Leica EZ4) at ×35 [48–52] in order to identify *S. scabiei* mites following morphological criteria [53].

### Histopathology, immunohistochemistry and inflammatory cell study

Skin sections 3–4 µm thick from both wildb1 and the control wild boar were stained with Mayer’s haematoxylin and eosin. Additional sections of the skin were stained with Toluidine blue. In the skin from wildb1 and control samples, eosinophil counts were performed on the haematoxylin and eosin staining, and mast cells were counted on the Toluidine blue staining. Additional paraffin 3-µm sections of the wildb1 and control wild boar skin samples were made for immunohistochemical analyses through specific staining to detect inflammatory cell types, namely macrophages, plasma cells, T cells and B cells. Immunohistochemical laboratory procedures were performed following the methodology described by Martínez and collaborators [54], allowing comparison with previously reported findings (Additional file 1: Table S1).

Eosinophils, mast cells, macrophages, plasma cells, T cells and B cells were counted at ×400 in five fields of each respective skin section staining from each wild boar (wildb1 and the control one), following the previously reported methodology [54]. The mean proportion of stained cells to total cells was averaged across the five fields. The cell counting was repeated twice by independent observers, and then the results were averaged. All the values (total counts, percentages and field means) were compared with Chi-square tests performed with R software version 4.0.2.

### Molecular analysis

Three wild boar mites (one from wildb1, one from wildb2 and one from wildb3) and 40 mites from 23 Iberian ibexes sympatric to these three wild boars from three different geographic areas were used for the genetic analysis (Additional file 2: Table S2, Fig. 2). The mites were individually isolated from frozen skin samples using the postponed isolation method [55, 56], and DNA was extracted following the HotSHOT plus ThermalSHOCK technique [57]. DNA was amplified through a multiplex 10× polymerase chain reaction (PCR), and multilocus genotyping using 10 specific *Sarcoptes* mite microsatellites as molecular markers was applied to determine the origin of the parasitic transmission [58]. The microsatellites were selected from the panel proposed by Walton et al. (2004) [12], namely selective androgen receptor modulators (SARMS) 33, 34, 35, 36, 37, 38, 40, 41, 44 and 45. A Bayesian assignment test was carried out to determine the most likely number of genetic clusters through the software STRUCTURE (v. 2.3.4) [59]. The lengths of burn-in period and number of Markov chain repetitions were 10,000 and 100,000, respectively. Twenty independent runs were performed for each K (for *K* = 1–10), using the admixture option as ancestry model. Selection of *K* was determined using the DK Evanno method [60]. Descriptive statistics, including multilocus proportion of shared alleles, was carried out with Microsatellite Analyser (MSA 4.1) [61], ignoring any previous clustering information. A consensus dendrogram was obtained using the neighbour-joining algorithm performing 1000 bootstraps in Population 1.2.32 software (https://bioinformatics.org/populations/) and then displayed by MEGA 4.1 (http://www.megasoftware.net).

## Results

### Post-mortem examination

Wildb1 had poor body condition and bilateral hair loss around the eyes, base of the ears, neck, scapular, humeral, axillar, inguinal and anterolateral femoral and tibia regions. On close examination, the skin was moderately thickened with small 1–2-mm orange pustules and crusts, especially on the neck (Fig. 1). Penetrating and exit traumatic injuries consistent with the gunshot were found in the abdominal and left scapular regions, respectively (Fig. 1).

The main internal findings were a general moderate enlargement of the peripheral lymph nodes and areas of reddening, consolidation and septal oedema in the right apical, middle and cranial aspect of the caudal (15%) lung lobes, consistent with a subacute to chronic cranioventral bronchopneumonia. Focally extensive white-yellow areas with increased texture of the caudal aspect of both caudal lung lobes, consistent with verminous pneumonia, were also present, accounting for less than 10% of the entire volume of the caudal lung lobes.

Both the skin scrapings and the KOH digestions allowed identification of mites that were morphologically consistent with *S. scabiei*.

### Histopathology, immunohistochemistry and inflammatory cell study

**Fig. 3:**
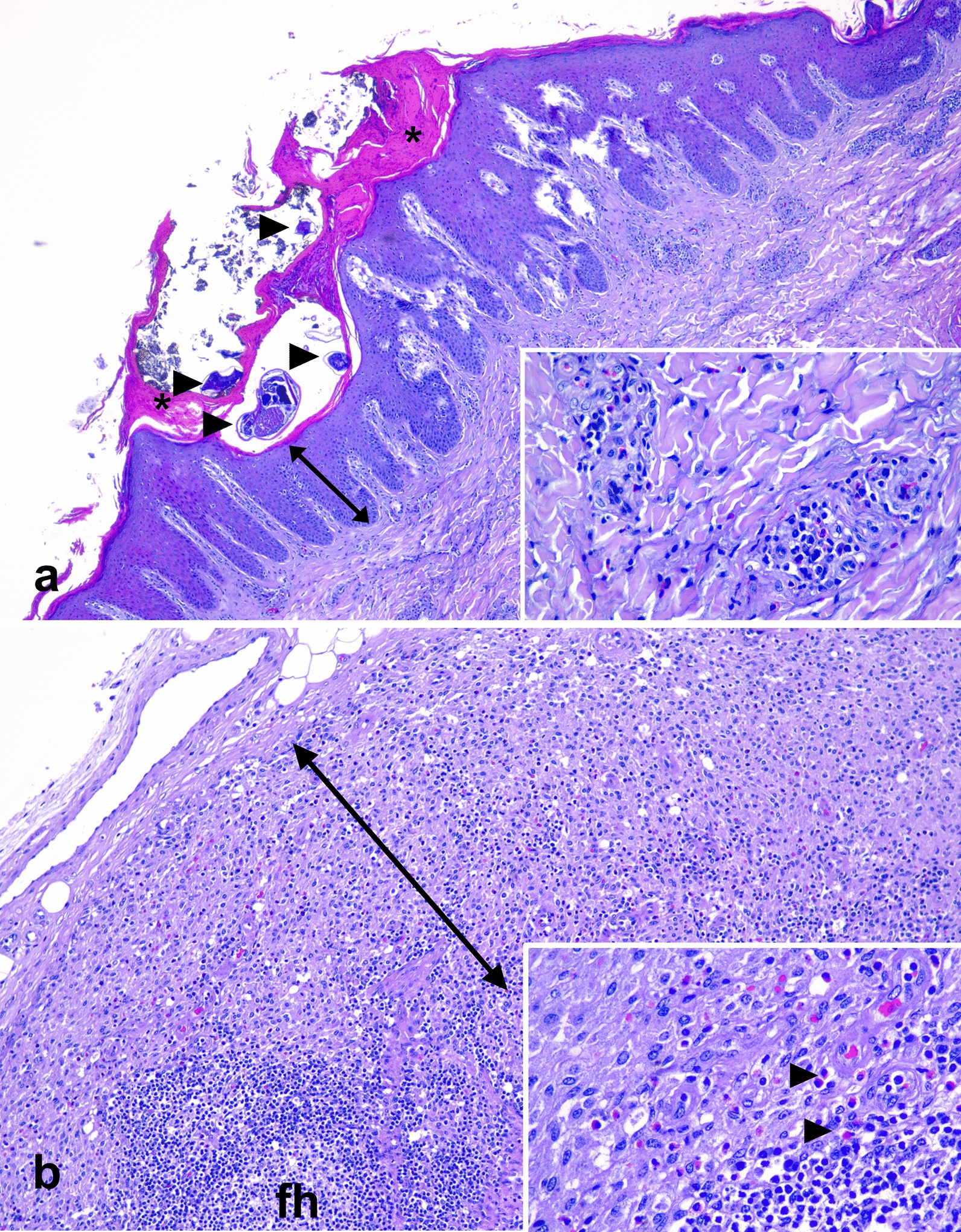
Microscopic lesions in a wild boar case with sarcoptic mange (wildb1). **a **Skin section from the ear. Cross section of *S. scabiei* and several eggs within a tunnel in the epidermis (arrowheads); note the hyperkeratosis (asterisks), the acanthosis and the deep rete ridges (arrow). **b** Regional lymph node with follicular hyperplasia (FH), formation of germinal centres, and subcapsular sinus expanded with macrophages and eosinophils (arrows in the inset). Haematoxylin and eosin stain

Approximately 5 cm of the lesions present in skin sections from perioral, eyelid, base of the ear and neck regions were examined. The parasitic burden was low on histology, and only a single tunnel with a female adult *S. scabiei* with three eggs in different stages of development could be seen in the section from the ear (Fig. 3). There was diffuse mild to moderate irregular hyperplasia of the epidermis with rete ridge formation and acanthosis, from 9 to 20 cell layers, and mild orthokeratotic hyperkeratosis, with focal areas of parakeratosis and occasional neutrophilic/eosinophilic intraepidermal pustules. In the dermis, a superficial to middle perivascular, mild to moderate, inflammatory infiltrate was present, formed mainly by mononuclear or lymphohistiocytic cells (macrophages and lymphocytes) with a variable ratio of eosinophils, which predominated in some areas. Loss of follicles or plugs of keratin within the follicles accounted for the macroscopic alopecia. Occasionally, only sebaceous glands surrounded by fibrous tissue remained, but sebaceous gland hyperplasia was not observed. In one of the sections, the lumen of a single follicle was dilated and filled with closely packed *Demodex* sp. In the cervical lymph nodes, macrophages and eosinophils filled and expanded the subcapsular sinuses.

In the lungs, the histopathological lesions observed matched those seen in the macroscopic examination. Suppurative chronic bronchopneumonia (neutrophils filling the lumen of bronchi, bronchioles and surrounding alveolae with a moderate bronchial epithelial hyperplasia and limphohistiocytic peribronchial infiltrate with mild bronchial-associated lymphoid tissue hyperplasia) in the cranial lobes, consistent with bacterial bronchopneumonia, and verminous pneumonia in the caudal lobes (sections of adult metastrongyles in the lumen of bronchi) were seen. Opportunistic *Escherichia coli* was identified in microbiological culture of lung tissue. No significant lesions were observed in the sections from the heart, kidney, spleen, brain, liver, skeletal muscle and tonsils.

**Fig. 4:**
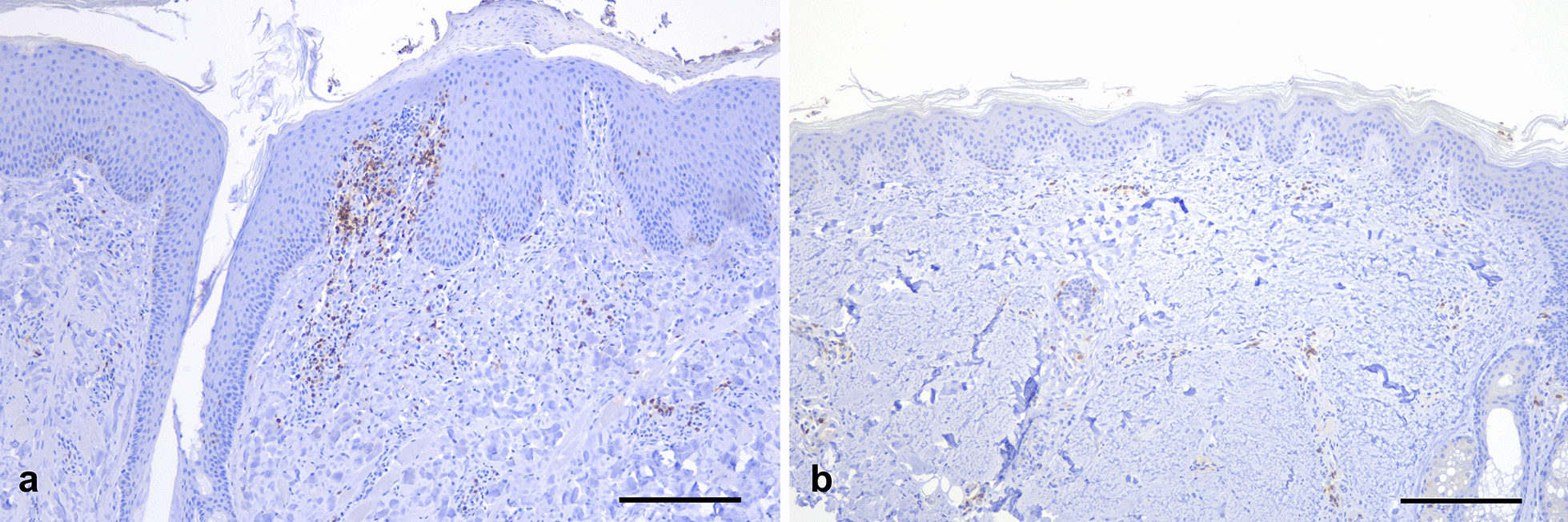
**a** Skin from wild boar case with sarcoptic mange (wildb1), positive immunolabelling in CD3 lymphocytes in the dermis and infiltrating the epidermis in focal areas with marked epidermal hyperplasia and hyperkeratosis. **b** Skin from a wild boar not affected by sarcoptic mange; bar = 200 μm

**Fig. 5:**
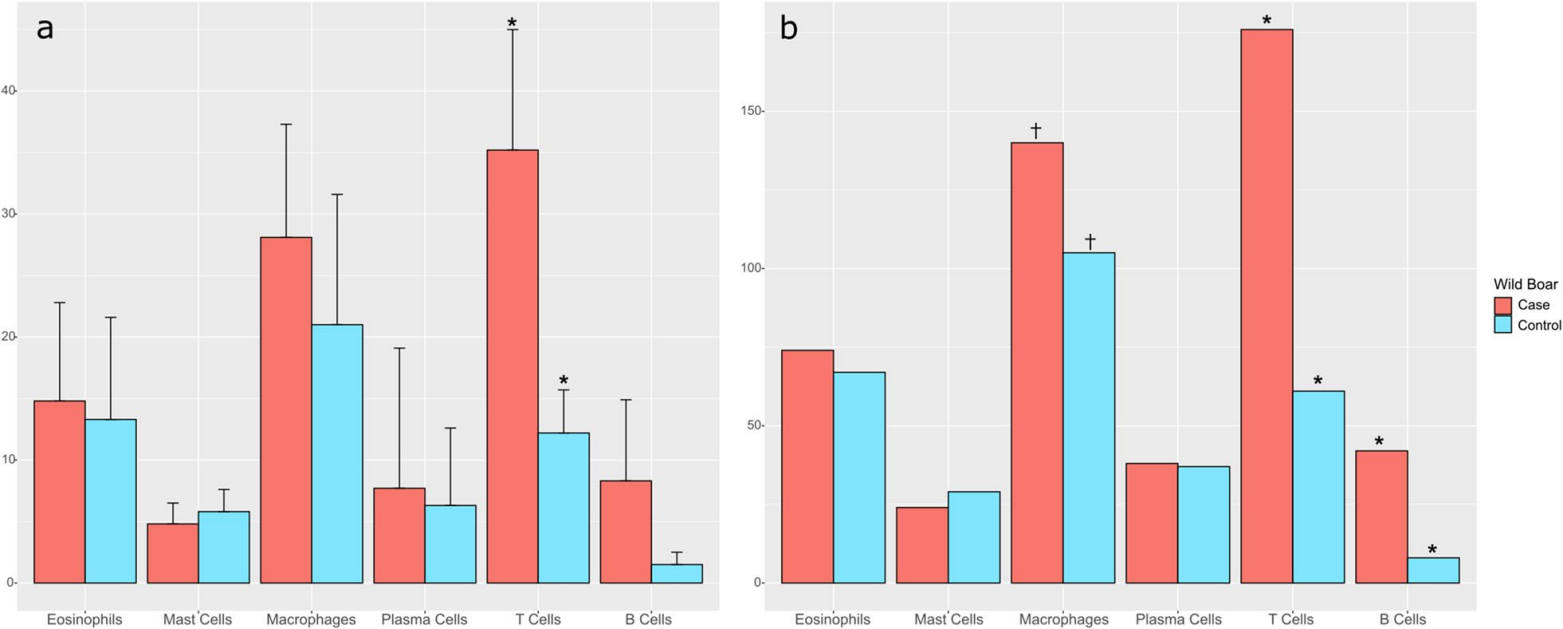
Comparison of the means (**a**) and the total cell counting (**b**) between the wild boar case of study (wildb1) and the control. †*P*-value < 0.05; *P-value < 0.001

Overall, wildb1 had significantly higher skin inflammatory infiltrate than the control wild boar (X^2^ = 6.189, *P*-value = 0.01285), with a higher absolute number of macrophages (X^2^ = 10.267, *P*-value = 0.001354), T cells (X^2^ = 111.6, *P*-value < 0.0001) and B cells (X^2^ = 47.184, *P*-value < 0.0001). Moreover, the mean of T cells per field (X^2^ = 11.16, *P*-value = 0.0008356) and the percentage of T cells (X2 = 4.4413, *P*-value = 0.03508) were also significantly higher in wildb1 than in the controls (Figs. 4 and 5).

### Molecular analysis

**Fig. 6:**
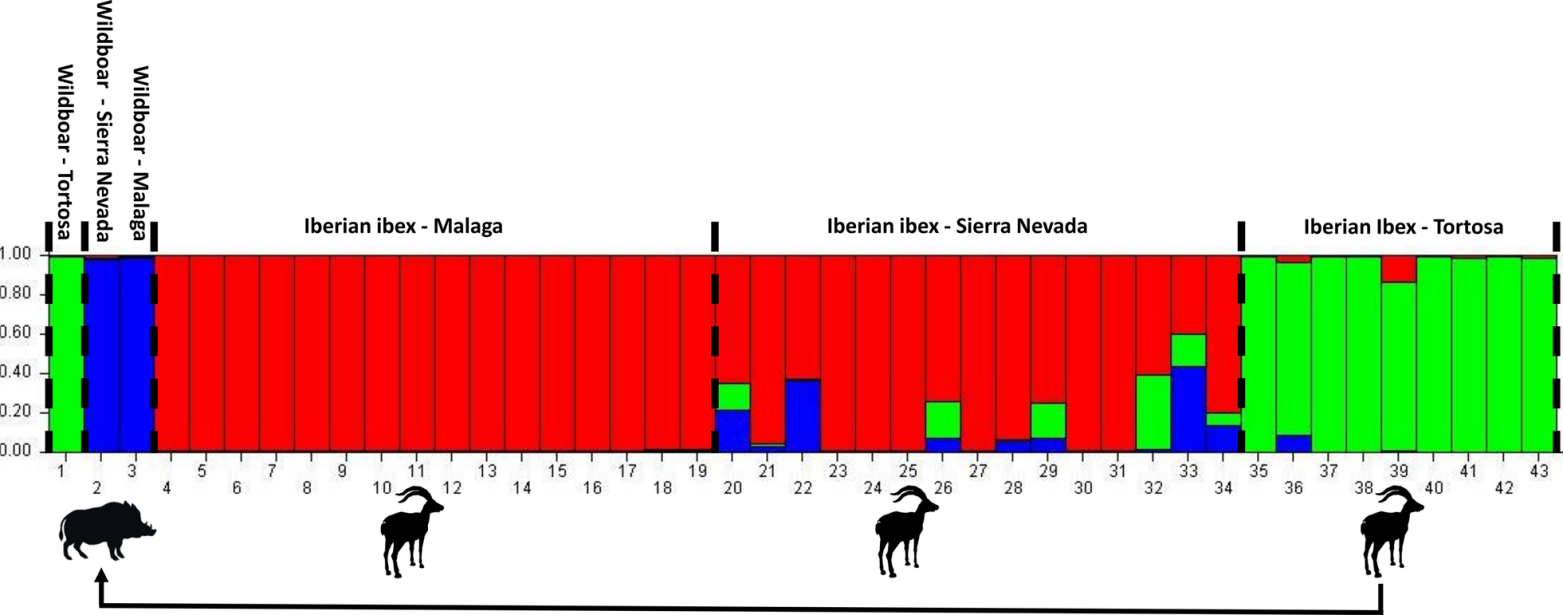
Bar plot generated with STRUCTURE 2.3.4 showing the three genetic mite clusters identified in the wild boars and Iberian ibex sampled. Each bar represents a *Sarcoptes* mite sample, and the height of each coloured segment is proportional to the membership fraction in each cluster

**Fig. 7:**
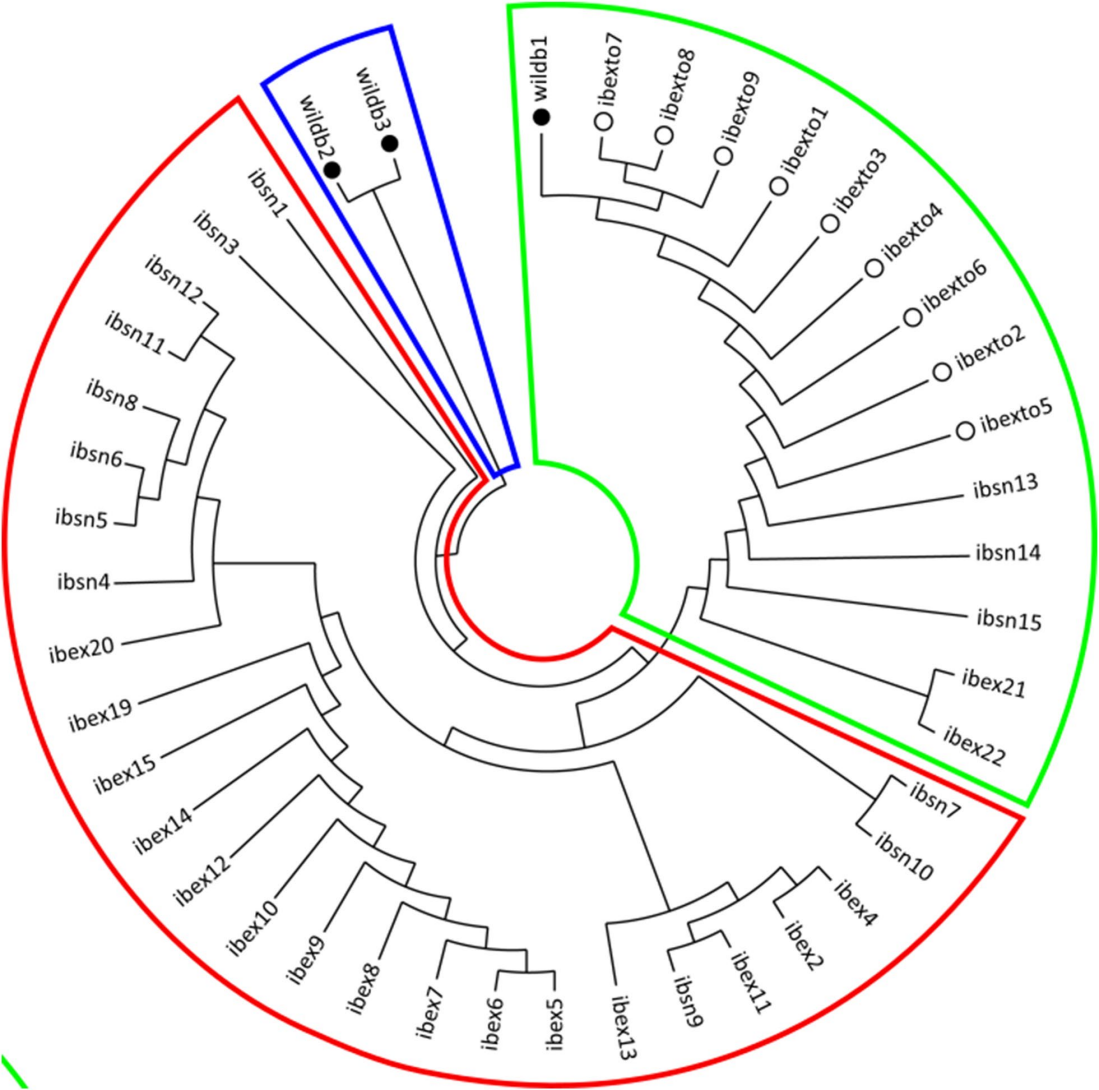
Neighbour-joining phylogenetic tree constructed by using distance matrices with Populations 1.2.32 and displayed with MEGA 4.1. The names of the samples are explained in Additional file 2: Table S2. Full circles represent wild boar-derived mites, while empty circles represent mites from ibexes from Tortosa. Colours are related to the membership clusters explained in Fig. 6

Forty alleles were obtained from 10 microsatellite loci, ranging from three (SARMS 33, 34, 37, 40, 41, 44) to six (SARMS 36) for each locus (Additional file 3: Table S3). Twenty-four private alleles were detected overall, ranging from nine (wildb3) to one (Ibexto), and no private alleles were detected in the wild boar from PTB and the ibexes from Málaga (Additional file 4: Table S4). The Bayesian assignment test for multilocus genotyping identified three main mite clusters (Fig. 6) obtained through the DK method (*K* = 3). The mite from wildb1 from Tortosa grouped with the mites from sympatric ibexes with 99% probability of belonging to that ancestry-inferred cluster, while wildb2 and wildb3 mites had 98 and 99% probability of belonging to the same cluster, respectively. Although assigned to the southern ibex cluster, the mites from ibexsn3 and ibexsn14 had 37% and 44% probability, respectively, of belonging to the wildb2 and wildb3 cluster. A consensus dendrogram tree showing the genetic distances between all the individual samples is shown in Fig. 7.

## Discussion

This study reports the first documented clinical cases of sarcoptic mange in wild boars from the Iberian Peninsula and, most importantly, the first interspecific transmission of *S. scabiei* from a wild ruminant host to wild boar. Cross-transmission of sarcoptic mange between different host species has been widely studied in experimental trials and spontaneous cases. Although sarcoptic mange infection is widespread in the Iberian ibex populations [39, 40, 52, 62–65], to date no cross-transmission of *S. scabiei* with wild boar has been reported. In addition, literature of sarcoptic mange in wild boars is scarce, contrasting with extensive descriptions in domestic pigs [7, 9, 32, 66, 67].

*Sarcoptes scabiei* is attributed a certain host specificity [5, 68, 69]. However, cross-transmissions between phylogenetically related species have been reported both experimentally, for example from domestic goats (*Capra aegagrus hircus*) to Cantabrian chamois (*Rupicapra pyrenaica parva*) [20], and spontaneously, for example from Cantabrian chamois to red deer (*Cervus elaphus*) and roe deer (*Capreolus capreolus*) [51, 70] or from Iberian ibex to red deer [71]. The success of *S. scabiei* cross-transmission events between phylogenetically distant species, for example between domestic pigs and ruminants, depends on the ecological fitting between the minimal resources required by the mite to survive and reproduce and those that the mite can find in the naïve host species [72, 73]. Such phylogenetically distant cross-transmission usually course as self-limiting infestations that heal when the mites supplied by the reservoir host species disappear because they cannot meet the aforementioned minimal requirements in the new host [15, 72, 73]. Nevertheless, cross-transmission of sarcoptic mange between genetically distant species may occur in prey–predator relationships, such as lion (*Panthera leo*) and wildebeest (*Connochaetes* sp.); cheetah (*Acinonyx jubatus*) and Thompson’s gazelle (*Eudorcas thomsonii*) [21]; and wolf (*Canis lupus*) and its prey herbivores [74]. In the present case, wildb1 could have acquired the mites from infected sympatric ibexes by scavenging the carcass of a recently dead mangy individual [75]. Alternatively, wildb1 could have been exposed to *S. scabiei* through direct or indirect contact with a dying mangy ibex. The mites from wildb2 and wildb3 clustered together and separately from those isolated in their sympatric Iberian ibexes, suggesting they were infected by a wild boar variant [7]. However, a greater number of mites from a range of all sympatric putative sources (e.g., foxes or wild lagomorphs) should have been analysed to support robustly any hypothesis on the origin of mites, including a potential mite exchange between Iberian ibexes, where sarcoptic mange is endemic [39, 62, 65], and wild boars.

Gene flow is commonly used as a proxy for population connectivity, and thus it can be considered a good indicator of *Sarcoptes* mite transmission between different host individuals, as it has been previously described in other wildlife species to understand the source of sarcoptic mange infection [68, 69]. The clustering of the mites from the two southern ibex populations separately from the mites from the northern ibex population agrees with a recent molecular epidemiological study [11]. The clustering and occurrence of multiple private alleles in the *Sarcoptes* mites from the southern wild boars (seven for wildb2 and nine in wildb3, Additional file 4: Table S4) suggests that they could be affected by specific omnivorous *S. scabiei* [10, 69] with little gene flow with other populations. However, since the numbers of wild boars studied and mites retrieved and analysed are low, further analyses of *S. scabiei* mites from wild boars in Spain are required to clarify the potential gene flow between mites affecting wild boars and the wild ruminant populations endemically affected by sarcoptic mange countrywide.

The combination of chronic skin lesions and bacterial bronchopneumonia observed in wildb1 has previously been reported in Mediterranean wild boars [67]. Sarcoptic mange skin lesions in domestic pigs usually include major epidermal changes, as acanthosis, rete peg hypertrophy, para-hyperkeratosis, necrosis, erosion, microabscesses, transudation, spongiosis and scales with bacterial colonies, piknotic neutrophils and occasional eosinophils. In the dermis, it distinctively causes oedema, vasculitis with extravasated erythrocytes, granulocytes (eosinophils) and diffuse monocyte infiltrates, with lymphoid cuffs around middle sized blood vessels, sebaceous gland hyperplasia and dilated acini of apocrine sweat glands in severe lesions [26, 76–78]. Compared to those, the lesions in wildb1 could be considered moderate, since no severe oedema, haemorrhages or vasculitis were seen in the dermis.

**Fig. 8:**
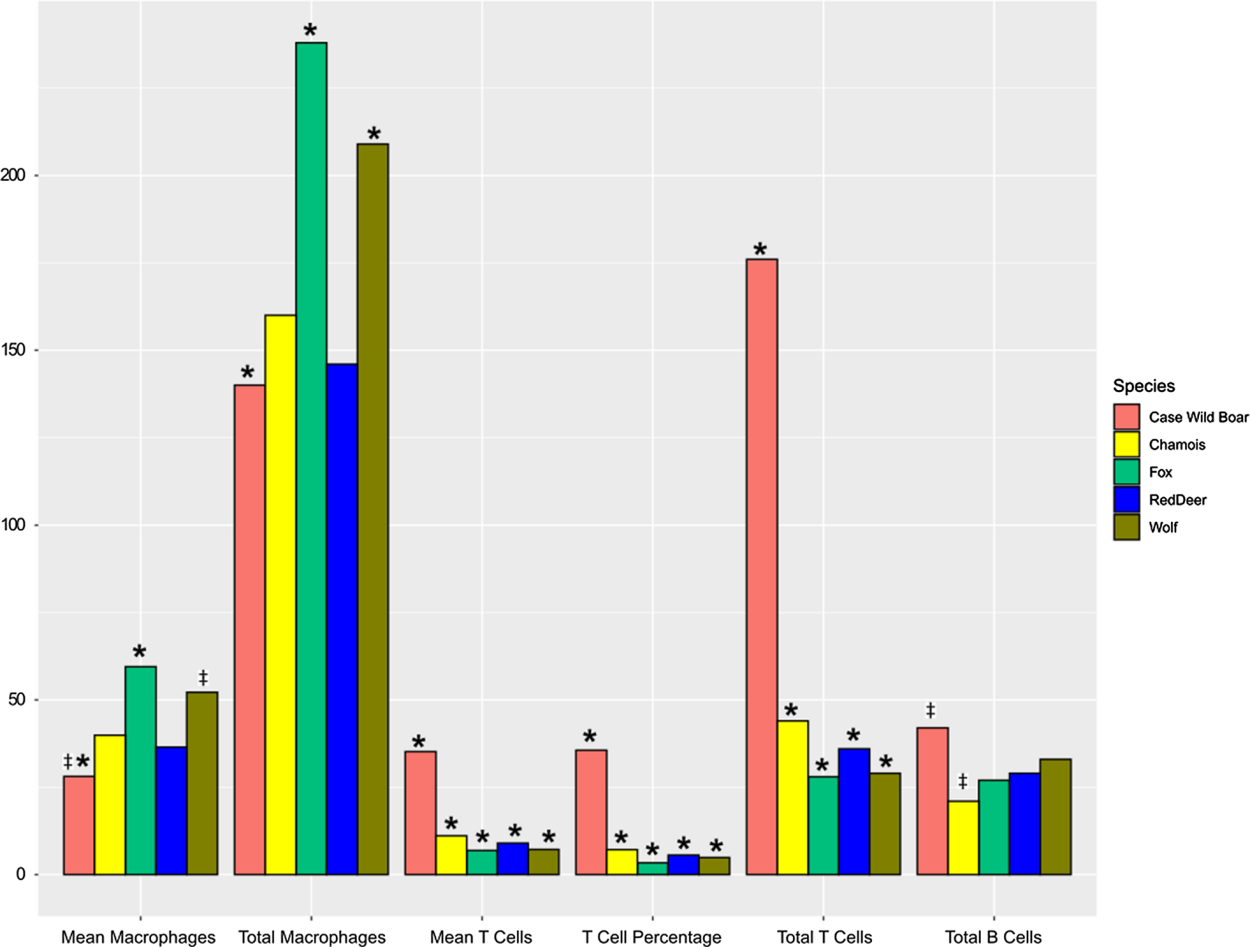
Inflammatory infiltrate cell population values statistically different between the wild boar case of study (wildb1) and the different species previously reported in Martínez et al. 2020. ‡*P*-value < 0.01; *P-value < 0.001

Wildb1 had apparently developed the typical inflammatory dermal reaction seen in domestic pigs with ordinary scabies, where the lymphocytes are the predominant cell (75%) [78]. Specifically, in domestic pigs, the adaptive immune reaction against *S. scabiei* consists in increasing the T cell infiltrate in the dermis following a perivascular pattern [77]. This T infiltrate was also higher in wildb1 than in other wildlife species infested by *S. scabiei*, such as Cantabrian chamois (X^2^ = 12.544, *P*-value = 0.0003974), red deer (X^2^ = 15.53, *P*-value < 0.0001), wolf (X^2^ = 18.491, *P*-value < 0.0001) and red fox (*Vulpes vulpes*) (X^2^ = 19.024, *P*-value < 0.0001) (Fig. 8) [54]. Wildb1 total B (X^2^ = 39.962, *P*-value < 0.0001) and plasma cells (X^2^ = 35.042, *P*-value < 0.0001) were also higher than in chamois. On the other hand, the wildb1 macrophage values were lower than in wolf and red fox (X^2^ = 11.255, *P*-value = 0.000794) (Fig. 8) [54]. Altogether, the pathological findings indicate that wildb1 had an ordinary scabies reaction type (adaptive immune response), which coincides not only with the cell type infiltrate, but also with the macroscopic lesions, the low number of mites found and the self-limiting nature of *S. scabiei* infections usually described in interspecific cross-transmissions. Contrastingly, scabietic wild boars probably infected with the host-specific *S. scabiei* strain have a mixed inflammatory infiltrate with eosinophils as the most abundant cell [44], which is consistent with an allergic and immediate hypersensitivity response, as observed in domestic pigs [77].

The clinical outcome of sarcoptic mange after interspecific cross-transmission does not depend only on the phylogenetic relationship between the host species, but also on the host immunity and status. This is shown by the different clinical course of each one of the three naïve roe deer that incidentally developed sarcoptic mange after being housed with a mangy chamois [23]. Therefore, wildb1 might have developed clinical mange partly because its own immune status or previous conditions, apart from exposure to the mite. Consequently, other wild boars could be carrying *S. scabiei* mites and spreading them in PTB while having just a mild or subclinical infection. Consequently, the surveillance and management of sarcoptic mange outbreaks should not only focus in the more severely affected species, but also cover other sympatric ungulates, in order to detect other complementary reservoir hosts.

Considering the lesions, the genetics and the ecological circumstances, this case of interspecific cross-transmission of sarcoptic mange could be attributed to: (1) the pressure of infection in the area; (2) the consumption of or contact with carcasses of severely infested Iberian ibex; (3) a state of immunosuppression of the wild boar, which would also fit the opportunistic *E. coli* growth in the lungs and the proliferation of *Demodex* sp.; and (4) a combination of the aforementioned factors.

## Conclusions

This study reports the first clinical cases of sarcoptic mange in wild boar in the Iberian Peninsula, and provides evidence of an interspecific cross-transmission event of *S. scabiei* from Iberian ibex to wild boar. Wild boars are suitable hosts for *S. scabiei* infection from sympatric herbivores, and the immune response, clinical course and associated macroscopic and histopathological lesions may vary according to the *S. scabiei* strain. Further investigations on the epidemiology and pathology of sarcoptic mange in wild boars wherever other endemically infected herbivore or carnivore populations are present seem desirable.

## Supplementary Information

**Additional file 1: Table S1.** Protocols used for the characterisation of inflammatory cell types in skin lesion samples.

**Additional file 2: Table S2.** Samples used for the genetic characterisation of *S. scabiei* mites.

**Additional file 3: Table S3.***Sarcoptes scabiei* alleles identified in the 10 microsatellite loci analysed in mites from wild boars and Iberian ibex from Spain.

**Additional file 4: Table S4.** Proteomics private alleles found per microsatellite locus in each sampled population of *S. scabiei* mites.

## Data Availability

The datasets generated during and/or analysed during the current study are available from the corresponding author on reasonable request.

## Abbreviations

KOH: Potassium hydroxide
Ibsn: Iberian ibex from Sierra Nevada
Ibexto: Iberian ibex from PTB
MSA: Microsatellite analyser
PCR: Polymerase chain reaction
PTB: Ports de Tortosa i Beseit Natural Park
wildb1: Wild boar from PTB, the study case
wildb2: Wild boar from Sierra Nevada
wildb3: Wild boar from Málaga
