# Supplementary material for: First report of interspecific transmission of sarcoptic mange from Iberian ibex to wild boar
Source: Parasit Vectors. 2021 Sep 19;14:481. doi: 10.1186/s13071-021-04979-w (PMC8451136; doi:10.1186/s13071-021-04979-w)
Supplement: Supplementary file 1 — Additional file 1: Table S1. Protocols used for the characterisation of inflammatory cell types in skin lesion samples. [file 13071_2021_4979_MOESM1_ESM.docx]

**Additional file 1: Table S1.** Protocols used for the characterization of inflammatory cell types in skin lesion samples.

| **Target cell type** | **Method** | **Reactive** |
| --- | --- | --- |
| **Eosinophil** | Haematoxylin and eosin staining |  |
| **Mast cell** | Toluidine blue staining |  |
| **Macrophage** | Immunohistochemistry,  Avidin-Biotin Complex  Method  (Vector Laboratories, CA, USA) | Antibody Iba1 (WAKO 019_19741),  rabbit polyclonal (1:1000)* |
| **Plasma Cell** | Immunohistochemistry,  Avidin-Biotin Complex  Method  (Vector Laboratories, CA, USA) | Antibody Lambda (Dako A0193),  rabbit polyclonal (1:1000)* |
| **Pan-T cell** | Immunohistochemistry,  Avidin-Biotin Complex  Method  (Vector Laboratories, CA, USA) | Antibody CD3 (Novocastra-CL-L-CD3-565),  mouse monoclonal (1:500)** |
| **Pan-B cell** | Immunohistochemistry,  Avidin-Biotin Complex  Method  (Vector Laboratories, CA, USA) | Antibody CD20 (ThermoFisher-PA516701),  rabbit polyclonal (1:200)* |

*Biotinylated Secondary Antibody Anti-rabbit (1:200)

**Biotinylated Secondary Antibody Anti-mouse (1:200)
