# Supplementary material for: First report of interspecific transmission of sarcoptic mange from Iberian ibex to wild boar
Source: Parasit Vectors. 2021 Sep 19;14:481. doi: 10.1186/s13071-021-04979-w (PMC8451136; doi:10.1186/s13071-021-04979-w)
Supplement: Supplementary file 2 — Additional file 2: Table S2. Samples used for the genetic characterisation of S. scabiei mites. [file 13071_2021_4979_MOESM2_ESM.docx]

**Additional file 2: Table S2.** Samples used for the genetic characterization of *Sarcoptes scabiei* mites.

| **Host species** | **Origin** | **Sample code** | **Animal** | **Mites** | **Year of collection** |
| --- | --- | --- | --- | --- | --- |
|  | Tortosa | wildb1 | 1 | 1 | 2018 |
| **Wild boar** | Sierra Nevada | wildb2 | 1 | 1 | 2014 |
|  | Málaga | wildb3 | 1 | 1 | 2017 |
|  | Tortosa | ibexto1-9 | 8 | 9 | 2014 |
| **Iberian ibex** | Sierra Nevada | ibsn1-15 | 8 | 15 | 2015 |
|  | Málaga | ibex4-15 and 19-22 | 7 | 16 | 2017 |
