# Supplementary material for: First report of interspecific transmission of sarcoptic mange from Iberian ibex to wild boar
Source: Parasit Vectors. 2021 Sep 19;14:481. doi: 10.1186/s13071-021-04979-w (PMC8451136; doi:10.1186/s13071-021-04979-w)
Supplement: Supplementary file 3 — Additional file 3: Table S3.Sarcoptes scabiei alleles identified in the 10 microsatellite loci analysed in mites from wild boars and Iberian ibex from Spain. [file 13071_2021_4979_MOESM3_ESM.docx]

**Additional file 3: Table S3.** *Sarcoptes scabiei* alleles identified in the ten microsatellite loci analysed in mites from wild boars and Iberian ibex from Spain.

N = Number of the corresponding allele identified for each locus.

| MS33 | | MS34 | | MS35 | | MS36 | | MS37 | | MS38 | | MS40 | | MS41 | | MS44 | | MS45 | |  |  |
| --- | --- | --- | --- | --- | --- | --- | --- | --- | --- | --- | --- | --- | --- | --- | --- | --- | --- | --- | --- | --- | --- |
| Alleles | N | Alleles | N | Alleles | N | Alleles | N | Alleles | N | Alleles | N | Alleles | N | Alleles | N | Alleles | N | Alleles | N |  |  |
| 226 | 78 | 176 | 23 | 130 | 1 | 265 | 1 | 170 | 2 | 207 | 1 | 215 | 80 | 224 | 2 | 262 | 79 | 178 | 2 |  |  |
| 242 | 2 | 212 | 1 | 146 | 2 | 273 | 1 | 172 | 82 | 211 | 1 | 217 | 2 | 234 | 57 | 266 | 1 | 194 | 3 |  |  |
| 274 | 2 | 214 | 51 | 162 | 17 | 275 | 1 | 198 | 2 | 213 | 1 | 225 | 2 | 236 | 27 | 268 | 2 | 196 | 61 |  |  |
|  |  | 216 | 7 | 164 | 64 | 279 | 69 |  |  | 215 | 79 | 229 | 2 |  |  |  |  | 198 | 17 |  |  |
|  |  |  |  |  |  | 281 | 4 |  |  | 217 | 2 |  |  |  |  |  |  | 200 | 1 |  |  |
|  |  |  |  |  |  | 283 | 2 |  |  |  |  |  |  |  |  |  |  |  |  |  |  |
