# Supplementary material for: First report of interspecific transmission of sarcoptic mange from Iberian ibex to wild boar
Source: Parasit Vectors. 2021 Sep 19;14:481. doi: 10.1186/s13071-021-04979-w (PMC8451136; doi:10.1186/s13071-021-04979-w)
Supplement: Supplementary file 4 — Additional file 4: Table S4. Proteomics private alleles found per microsatellite locus in each sampled population of S. scabiei mites. [file 13071_2021_4979_MOESM4_ESM.docx]

| **Additional file 4: Table S4.** Private alleles found per microsatellite locus in each sampled population of *Sarcoptes scabiei* mites | | | | | | | | | |  |  |
| --- | --- | --- | --- | --- | --- | --- | --- | --- | --- | --- | --- |
|  | **Sarms33** | **Sarms34** | **Sarms35** | **Sarms36** | **Sarms37** | **Sarms38** | **Sarms40** | **Sarms41** | **Sarms44** | **Sarms45** | **Total** |
| **wild_tortosa** |  |  |  |  |  |  |  |  |  |  | **0** |
| **wild_sn** | 242 (2) |  | 130 (1) | 283 (2) | 198 (2) | 217 (2) | 229 (2) |  | 226 (1) |  | **7** |
| **wild_malaga** | 274 (2) |  | 146 (2) | 275 (1) | 170 (2) | 207 (1), 211 (1) |  | 224 (2) | 268 (2) | 178 (2) | **9** |
| **ibex_malaga** |  |  |  |  |  |  |  |  |  |  | **0** |
| **ibex_sn** |  | 212 (1) |  | 273 (1), 265 (1) |  | 213 (1) | 217 (2), 225 (2) |  |  | 194 (3) | **7** |
| **ibex_to** |  |  |  |  |  |  |  |  |  | 200 (1) | **1** |
| **Total** | **2** | **1** | **2** | **4** | **2** | **4** | **3** | **1** | **2** | **3** | **24** |
